# Distributional analyses reveal the individual differences in congruency sequence effect

**DOI:** 10.1371/journal.pone.0272621

**Published:** 2022-08-22

**Authors:** Dandan Tang, Xuefei Chen, Hong Li, Yi Lei

**Affiliations:** 1 School of Teacher Education, Zunyi Normal University & Zunyi Institute of Family Education, Zunyi, Guizhou, China; 2 College of Teacher Education, Qujing Normal University, Qujing, Yunnan, China; 3 Institute of Brain and Psychological Sciences, Sichuan Normal University, Chendu, China; Centre National de la Recherche Scientifique, FRANCE

## Abstract

As a sequential modulation of conflict, congruency sequence effect indexes a conflict-induced performance improvement, which is observed as reduced congruency effects for trials after the incongruent trials than for trials after the congruent trials. Although congruency sequence effect has been investigated widely in healthy humans, the studies of distributional characteristics across prototypical congruency tasks are scarce. To investigate this issue, the present study adopts the between-subjects design to carry out three experiments, where subjects were separately informed to perform the Stroop, word Flanker, and letter Flanker tasks. The results showed that congruency sequence effect occurred in the congruent and incongruent trials in the Stroop and word Flanker tasks, respectively, and absented in the letter Flanker task, which is interpreted as the differences in the nature and difficulty of the tasks. The distributional properties of congruency sequence effect did not significantly differ from the Gaussian distribution in the Stroop and word Flanker tasks, but not in the letter Flanker task, suggesting the inter-individual variability of congruency sequence effect depends on the nature of tasks. Importantly, the delta plot analyses showed pronouncedly increased congruency sequence effect over the slowest percentile bines in both the Stroop and word Flanker tasks, verifying the activation suppression hypothesis. Altogether, the present study enriches the literature on the distributional characteristics of congruency sequence effect.

## Introduction

The incongruence relative to congruence between target and distractor in the congruency tasks [1], e.g., the Stroop task [2], Flanker task [3], and Simon task [4], usually results in longer response times (RTs) and higher error rates in human. Such incongruence activates the cognitive control system to recruit the mechanisms of cognitive control to improve performance [5–9]. As a typically sequential modulation of performance, congruency sequence effect, which is thought to encompass two basic cognitive processes: conflict monitoring and control implementation [7, 8, 10], denotes such a conflict-induced performance improvement, which is observed as reduced congruency effects in the current trials after an incongruent trial as compared with a congruent trial in the congruency tasks [11–19]. The congruency effects are usually defined as the performance differences between the incongruent and congruent trials.

For example, in the Stroop task, stimuli comprise words with congruent or incongruent semantics with the printed color (e.g., BLUE is printed in blue font for the congruent condition, or RED is printed in blue font for the incongruent condition). Participants are informed to respond to the printed color and to ignore the semantics of the words. And in the letter Flanker task, stimuli consist of strings of horizontal-arranged letters with a central target letter compatible or incomparable with the flanker letters. Participants are informed to respond to the target letter and to ignore the flanker letters. The congruency effects can be indexed by longer RTs and/or higher error rates in the incongruent (I) relative to congruent (C) trial, which are calculated by incongruent minus congruent (I-C) in RTs or error rates. The congruency sequence effect can be quantified by the following formula: congruency sequence effect = RTs_[(cI–cC)–(iI–iC)]_ [20, 21], where cC is a congruent trial followed by a congruent trial, cI is a congruent trial followed by an incongruent trial, iC is an incongruent trial followed by a congruent trial, and iI is an incongruent trial followed by an incongruent trial. Accordingly, congruency sequence effect is revealed by smaller congruency effects in the current trials preceded by an incongruent relative to congruent trial, i.e., RTs_(iI–iC)_ < RTs_(cI–cC)_ [11, 14, 21–23].

The conflict monitoring theory [5, 10] proposes a top-down attentional control explanation of congruency sequence effect, which supposes that congruency sequence effect stems from a conflict-driven continuously adjustment in cognitive control [8, 14, 24–27]. This assumption has been supported by the neurophysiological data. That is, a conflict monitoring system (anterior cingulate cortex, ACC) on the medial surface of the frontal lobes of the brain monitors for the occurrence of conflict [5, 28–31]. Subsequently, the ACC exerts an influence on the dorsolateral prefrontal cortex (DLPFC), which triggers strong cognitive control processes and results in a behavioral adjustment [32–35].

Recently, individual differences in congruency sequence effect in patients with Parkinson’s disease [36, 37] and traumatic brain injury [38] or in healthy human have been studied [39–41]. In these studies, the behavioral data revealed comparable size of the congruency sequence effect between the experimental and control groups across the Stroop and Flanker tasks. Specifically, Wang et al. evaluated the regional homogeneity (ReHo) of resting-state functional magnetic resonance imaging (RS-fMRI) signals to explore the neural basis of individual differences in congruency sequence effect in the letter Flanker task [42]. They found significant positive correlations between the size of congruency sequence effect and the left DLPFC and left ventrolateral prefrontal cortex (VLPFC) ReHo values, suggesting that participants exhibiting greater ReHo in these regions tended to display a greater degree of congruency sequence effect. Importantly, they found that the ReHo values in the DLPFC predicted behavioral performance related to congruency sequence effect. Then, Wang et al. employed a psychophysiological interaction (PPI) analysis within the Stroop task to examine the functional connectivity mechanisms underlying the individual differences in congruency sequence effect [43]. The results found that high congruency sequence effect participants showed higher intra-CEN (central executive network) connectivity and lower intra-SN (salience network) connectivity; while low congruency sequence effect participants showed higher intra-SN connectivity and lower intra-CEN connectivity. Another study had reported that different activations in the prefrontal regions reflected the individual differences in congruency sequence effect [44]. Therefore, these studies reveal the underlying neural mechanisms of individual differences related to congruency sequence effect.

However, congruency sequence effect is traditionally obtained from the across-trial average of RTs_[(cI–cC)–(iI–iC)]_, resulting in (1) the inter-trial differences in congruency sequence effect are not detectable, and (2) the true effects are counteracted. In this case, the analysis of congruency sequence effect in single-trial level can provide the information on the inter-trial variability of congruency sequence effect and can help us to observe the true effects when assessing the inter-individual differences of congruency sequence effect. Usually, the delta plotanalysis can be used to investigate the inter-trial variability of the congruency effects and to reveal the inter-individual differences in the congruency effects [45–48]. However, it is unclear how congruency sequence effect varies with the RTs in the delta plot analysis. In addition, the Gaussian distribution model may demonstrate typical inter-individual differences in a data set. However, it is unclear if the distributional properties of congruency sequence effect significantly differ from Gaussian distribution across tasks.

Thus, the present study is designed to investigate the distributional characteristics of congruency sequence effect in healthy human across the Stroop [2], word Flanker [49, 50], and letter Flanker tasks [3] in three Experiments, and to reveal both the inter-trial and the inter-individual variability of congruency sequence effect. In Experiment 1, 122 heathy human participants were informed to perform the typical Stroop task [2]. In Experiment 2, 120 heathy human participants were informed to perform the word Flanker task [49, 50]. In Experiment 3, 124 heathy human participants were informed to perform the letter Flanker task [3]. In all three experiments, the proportion of the congruent and incongruent trials was equal; the confounding of both the bottom-up repetition effects [51, 52] and negative priming effects [53] was excluded. In this way, we expected to obtain a pure congruency sequence effect. Meanwhile, the delta plots [54] were adopted to examine how congruency sequence effect evolves with the RT distributions. We also would fit the Gaussian distribution to the RTs_[(cI–cC)–(iI–iC)]_ data to reveal the distributional patterns of congruency sequence effect. Although the variability of individual differences in congruency sequence effect had been observed in healthy human across the Stroop and Flanker tasks [39–43], finding the inter-trial and inter-individual variability of the congruency sequence effect across these congruency tasks in a study may provide evidence for the generality and robustness of the observations.

It is notable that in the Stroop task, the word reading and color naming are processed in the different processing pathway and the word reading shows a processing advantage over the color naming [55, 56] according to the parallel distributed processing model [57–59]. On the contrary, in the word Flanker and letter Flanker tasks, both target and flankers are processed in the same pathway and, therefore, the two dimensions always overlap with each other and the flanker dimension has no processing advantage over the target dimension according to the dimensional overlap theory [60, 61]. Additionally, the stimuli consist of Chinese characters and letters in the word Flanker and letter Flanker tasks, respectively. In this case, the nature of tasks and the processes of stimuli are different in all three tasks. Thus, we forecast that the pattern of congruency sequence effect will vary across tasks. In the Stroop task, congruency sequence effect will be determined by the congruent condition according to the previous reports [21, 22]; in the word Flanker and letter Flanker tasks, the patterns of congruency sequence effect may differ from that in the Stroop task due to the differences in the nature of task. That is, congruency sequence effect may be determined by the incongruent condition or may be absent in the word Flanker and letter Flanker tasks. In addition, we expect the Gaussian model will well fit the RT distributional characteristics of congruency sequence effect. According to the activation suppression hypothesis [48], we predict that congruency sequence effect increases over the slowest percentile bines. Thus, the present study findings will provide substantial evidence verifying both the inter-trial and the inter-individual variability of the congruency sequence effect for the first time.

## Experiment 1

### Method

#### Participants

122 healthy right-handed volunteers (45 women), between 18 and 26 years old (19.99±1.19, mean±SD, similarly hereinafter), took part in the experiment. All volunteers reported normal or corrected-to-normal vision and normal color perception. All volunteers provided written informed consent and were paid for their participation. The Zunyi Normal College Human Ethics Committee (Zunyi, China) approved the experimental procedure, which were in accordance with the standards of the Declaration of Helsinki. In addition, the volunteers were blind to the experimental design.

#### Apparatus, stimuli, and task

The stimuli were presented in white against a black background using E-Prime 2.0 software (Psychology Software Tools, Inc., Learning Research and Development Center, University of Pittsburgh) on a 17-in Lenovel computer monitor. Responses were registered using a standard QWERTY keyboard. Participants sat approximately 60 cm away from the screen.

The stimuli consisted of four words (RED, GREEN, YELLOW, and BLUE; in Chinese with Song Ti font, size 28) that were printed in a red, green, yellow, or blue font. The RGB values for the stimulus colors were 255, 0, 0 (red); 0, 255, 0 (green); 255, 255, 0 (yellow); and 0, 0, 255 (blue), respectively. For the congruent condition, the color and word matched (e.g., the word “GREEN” was in green font); for the incongruent condition, the color and the word didn’t match (e.g., the word “RED” was in green font).

Participants were instructed to response to the printed color of word as quickly and accurately as possible, and to ignore the semantics of word. They were instructed to press the “D” key using the left middle finger if the color of the word was red, the “F” key using the left forefinger if the color of the word was green, the “J” key using the right forefinger if the color of the word was yellow, and the “K” key using the right middle finger if the color of the word was blue. The rules of stimulus-response button mapping were counterbalanced across the participants.

#### Procedure and design

**Fig 1:**
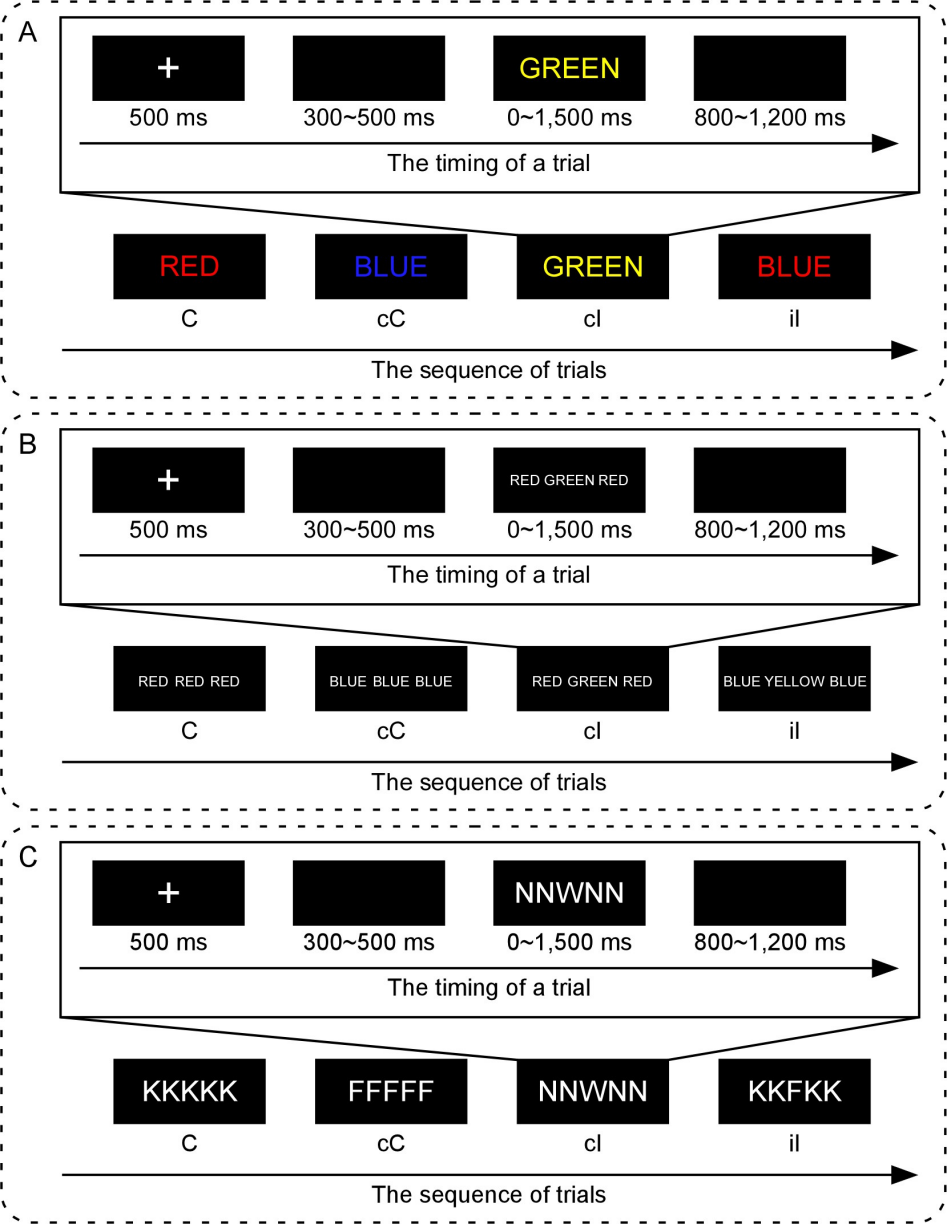
Experimental procedure for Experiments 1, 2, & 3. A probable pseudo-random sequence of four trials (bottom) and the detailed timing of one trial (top) are illustrated for Experiments 1, 2, & 3. Panel **A** shows the experimental procedure of the Stroop task in Experiment 1. Panel **B** shows the experimental procedure of the word Flanker task in Experiment 2. Panel **C** shows the experimental procedure of the letter Flanker task in Experiment 3. Note, in both Experiments 1 & 2, the words (“RED”, “GREEN”, “YELLOW”, and “BLUE”) are presented in Chinese with Song Ti font.

The experimental procedure is illustrated in Fig 1A. In each trial, the stimuli were presented as follows: (1) a white fixation ‘+’ for 500 ms; (2) a blank interval for 300–500 ms (the interval varied randomly); (3) a colored word until a key was pressed or for 1.5 s if there was no response registered; and (4) a blank interval for 800–1200 ms (interval varied randomly). Presentation order of the trials was pseudo-randomized by using experimental manipulation, which excluded the confounding of bottom-up repetition effects [51, 52] including the stimulus-stimulus, stimulus-response, and response-response repetitions. And only the trial sequences with the stimulus-stimulus, stimulus-response, and response-response changes were remained.

Participants performed a block of sixty-four practice trials prior to the completion of the formal experiment, which acquainted them with the experimental stimulus-response mapping. In the formal experiment, 576 trials were separated into four blocks of 144 trials each. In each experimental block, each of the congruent and incongruent conditions contained 72 trials. To assess congruency sequence effect, the stimuli were separated into the cC, cI, iC, and iI conditions according to the congruency (‘c’ was congruent, ‘i’ was incongruent) of the previous trial and that of the current trial. Each of the cC, cI, iC, and iI conditions contained 36 trials for each experimental block. There was a 2-minute break between blocks to minimize the effect of fatigue.

### Results

**Fig 2:**
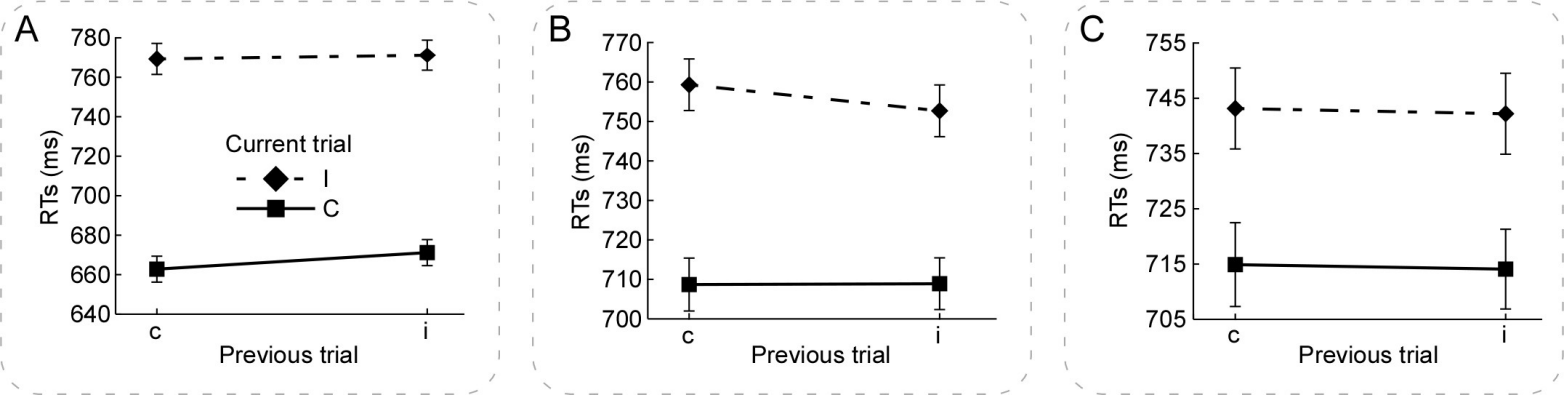
The mean RTs in Experiments 1, 2, & 3. Panels **A, B, & C** illustrate the mean RTs in the current trial, which was calculated according to the congruency (congruent, incongruent) in the previous and current trials in Experiments 1, 2, & 3, respectively. Panel **A** shows a significant congruency sequence effect in the current congruent trials in Experiment 1. Panel **B** shows a significant congruency sequence effect in the current incongruent trials in Experiment 2. Notably, the RTs_(iI–iC)_ was significantly lower than the RTs_(cI–cC)_ in Experiments 1 and 2. Panel **C** shows a non-significant congruency sequence effect in Experiment 3. For each condition, error bars represent ±SEM across the participants. NB. ‘c’ and ‘C’ are congruent condition, ‘i’ and ‘I’ are incongruent condition, RTs is response times.

The RTs and error rates were analyzed using the IBM SPSS Statistics 20.0 software. For the analysis of the RTs, all incorrect trials (5.86% of all trials), post-error trials (5.23% of all trials), and RT outliers (±3 SDs, 0.76% of all trials) were excluded. The general linear model (GLM) was used to compare the mean RTs for each condition. The two-way repeated-measures analysis of variance (ANOVA) was conducted for the mean RTs with the following variables: the congruency (congruent, incongruent) in the previous trials and the congruency (congruent, incongruent) in the current trials, to reveal the essence of congruency sequence effect. The significance level (*p*-value) was corrected using a Bonferroni method to control for the problem of multiple comparisons. Fig 2A illustrates the mean RTs in the current trials. Table 1 (the left column) shows the two-way repeated-measures ANOVA results for the mean RTs.

**Table 1:** Test statistics for the mean RTs in Experiments 1, 2, & 3.

The main effect of congruency in the previous trials (congruent, incongruent)								
Experiment 1			Experiment 2			Experiment 3		
*F*(1,121)	*p*	*η*_*p*_^2^	*F*(1,119)	*p*	*η*_*p*_^2^	*F*(1,123)	*p*	*η*_*p*_^2^
17.80	< .001	.128	7.90	.006	.062	<1	.484	.004
The main effect of congruency in the current trials (congruent, incongruent)								
Experiment 1			Experiment 2			Experiment 3		
*F*(1,121)	*p*	*η*_*p*_^2^	*F*(1,119)	*p*	*η*_*p*_^2^	*F*(1,123)	*p*	*η*_*p*_^2^
1088.86	< .001	.900	780.31	< .001	.868	236.14	< .001	.659
The interaction between the two factors								
Experiment 1			Experiment 2			Experiment 3		
*F*(1,121)	*p*	*η*_*p*_^2^	*F*(1,119)	*p*	*η*_*p*_^2^	*F*(1,123)	*p*	*η*_*p*_^2^
4.57	.035	.036	8.27	.005	.065	< 1	.960	< .001
Post hoc test (Bonferroni-corrected)								
Experiment 1				Experiment 2				
cC < iC, *p* < .001				iI < cI, *p* < .001				
cC < cI, *p* < .001				cC < cI, *p* < .001				
iC < iI, *p* < .001				iC < iI, *p* < .001				

Typically, the repeated-measures ANOVAs are based on the average of each condition for each participant. In this case, the inter-trial variability is completely blinded to the estimation of the significance of congruency sequence effect. Thus, to reveal the inter-trial variability of congruency sequence effect and to test the random factors, we used the linear mixed models to compare the RTs on the single-trial level of each condition of each participant, which included the congruency (congruent, incongruent) in both the previous and current trials as the fixed effects, and the gender (female, male) and age (continuous variable) as the random factors. The Bonferroni method was used to correct the significance level (*p* value) when carrying out the multiple comparisons procedure. The results are showed in Table 2 (the left column). It is notable that the results of the single-trial RTs are in accord with those of the mean RTs.

**Table 2:** Test statistics for the RTs on the single-trial level of each condition of each participant in Experiments 1, 2, & 3.

The main effect of congruency in the previous trials (congruent, incongruent)					
Experiment 1		Experiment 2		Experiment 3	
*F*(1,64024.45)	*P*	*F*(1, 62403.19)	*p*	*F*(1, 62446.01)	*p*
19.37	< .001	5.35	.021	<1	.619
The main effect of congruency in the current trials (congruent, incongruent)					
Experiment 1		Experiment 2		Experiment 3	
*F*(1, 64024.49)	*P*	*F*(1, 62403.23)	*p*	*F*(1, 62446.00)	*p*
4902.81	< .001	1285.49	< .001	335.78	< .001
The interaction between the two factors					
Experiment 1		Experiment 2		Experiment 3	
*F*(1, 64024.14)	*P*	*F*(1, 62403.13)	*p*	*F*(1, 62818.89)	*p*
4.75	.029	7.01	.008	< 1	.878
Pair-wise comparisons (Bonferroni-corrected)					
Experiment 1			Experiment 2		
cC < iC, *p* < .001			iI < cI, *p* < .001		
cC < cI, *p* < .001			cC < cI, *p* < .001		
iC < iI, *p* < .001			iC < iI, *p* < .001		

To demonstrate the reduced congruency effects in the current trial preceded by the incongruent compared with congruent trials, we calculated the differences of mean RTs in the current trials according to the congruency (c, i) in the previous trials, i.e., RTs_(iI–iC)_ and RTs_(cI–cC)_. And then, the RTs_(iI–iC)_ and RTs_(cI–cC)_ were compared within individuals using the paired-samples T test (2-tailed, Bonferroni correction, similarly hereinafter). The key observation revealed that RTs_(iI–iC)_ (100.01±36.90) was significantly smaller than RTs_(cI–cC)_ (106.50±39.85), *t*(121) *= -*2.14, *p* = .035, Cohen’s *d* = .176 [62].

**Fig 3:**
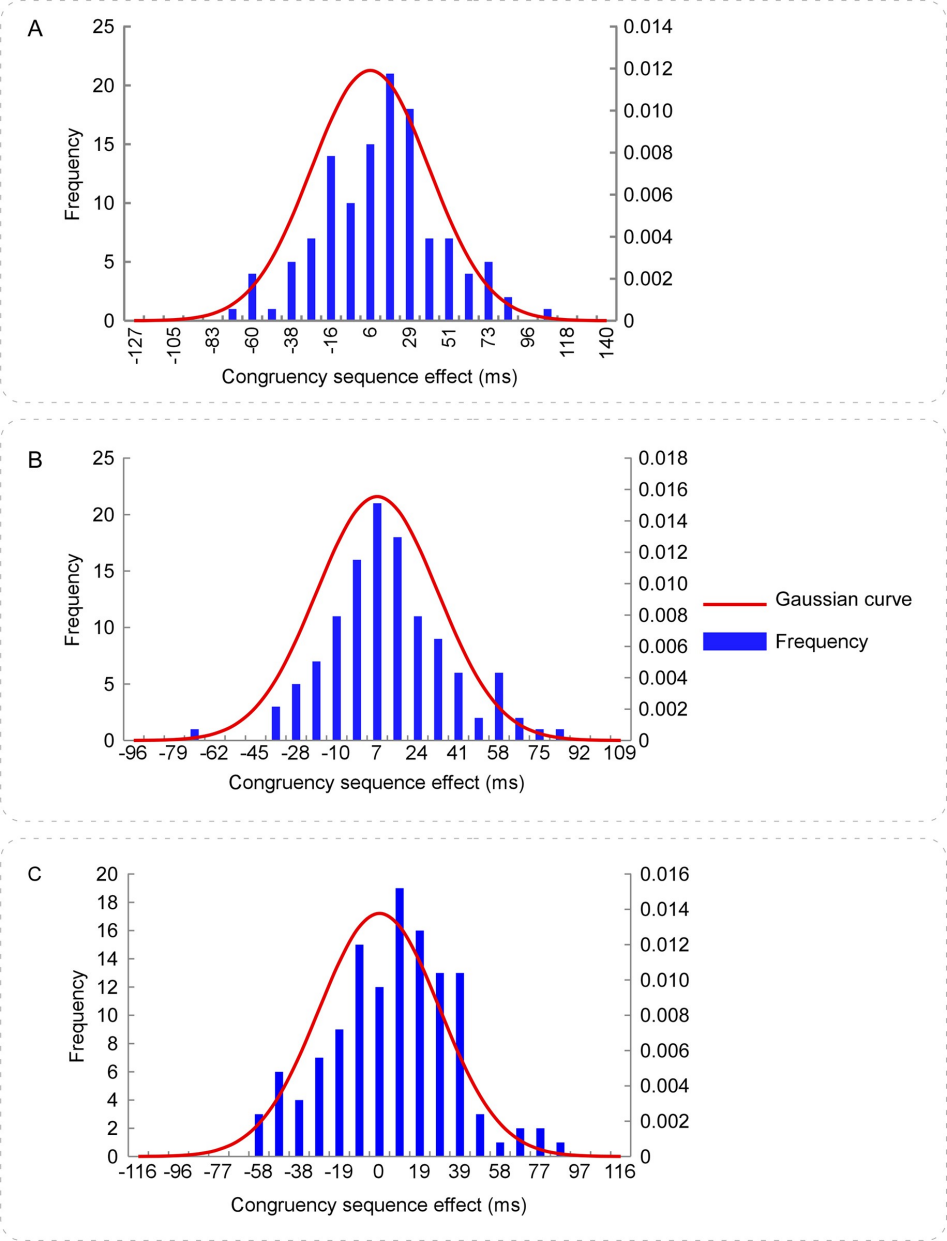
The frequency distribution of congruency sequence effect in the Stroop, word Flanker, and letter Flanker tasks. Panels **A, B, & C** illustrate the frequency distribution of congruency sequence effect in Experiments 1, 2, & 3, respectively. The size of congruency sequence effect was calculated by the RTs_[(cI–cC)–(iI–iC)]_. In each Experiment, the sizes of congruency sequence effect were divided into twenty-five bins according to the Gaussian distribution model.

**Fig 4:**
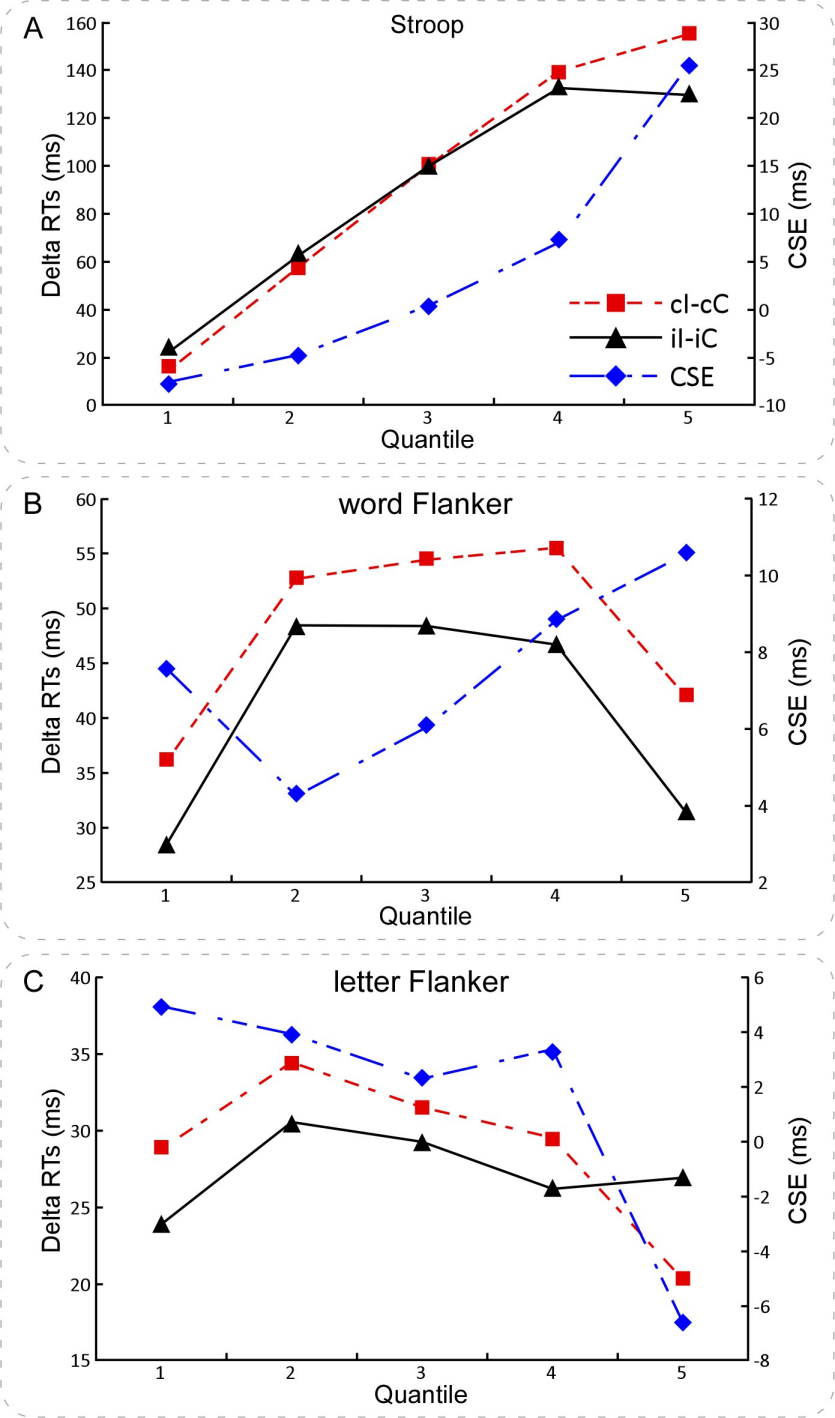
The RT distributions of congruency sequence effect in the Stroop, word Flanker, and letter Flanker tasks. Panels **A, B, & C** illustrate the time courses of congruency sequence effect in Experiments 1, 2, & 3, respectively. The congruency sequence effect, RTs_(cI–cC)_, and RTs_(iI–iC)_ were calculated over five quantiles in RTs of each participant and condition. Panel **A** depicts the time course of congruency sequence effect in the Stroop task. Noteworthy is that congruency sequence effect, RTs(_cI–cC)_, and RTs_(iI–iC)_ increased across all five quantiles. Panel **B** depicts the time course of congruency sequence effect in the word Flanker task. The negative slope between the first two quantiles indicated the decreased congruency sequence effect over the fastest bins; but the positive slope in the last three quantiles indicated the increased congruency sequence effect over the slowest bins. The positive slope between the first two quantiles indicated the increased congruency effects in RTs_(cI–cC)_ and RTs_(iI–iC)_ over the fastest bins; but the negative slope between the last two quantiles indicated the decreased congruency effects in RTs_(cI–cC)_ and RTs_(iI–iC)_ over the slowest bins. Panel **C** depicts the time course of congruency sequence effect in the letter Flanker task. The congruency sequence effect decreased with the increasing mean RTs, which was most pronounced between the last two quantiles. The RTs_(cI–cC)_ increased between the first two quantiles, but decreased in the last three quantiles. The RTs_(iI–iC)_ increased between the first two quantiles, but kept stable in the last three quantiles. NB. ‘cC’ and ‘iC’ are congruent condition preceded by congruent and incongruent conditions, respectively; ‘cI’ and ‘iI’ are incongruent condition preceded by congruent and incongruent conditions, respectively; RTs is response times; and CSE is congruency sequence effect calculated by the RTs_[(cI–cC)–(iI–iC)]_.

The size of congruency sequence effect was calculated according to the following formula (similarly hereinafter) [20, 21], congruency sequence effect = RTs_[(cI–cC)–(iI–iC)]_. To test the distributional properties of congruency sequence effect across participants, we performed the normality test by using the Shapiro-Wilk Test for RTs_[(cI–cC)–(iI–iC)]_. The results did not show significant difference from Gaussian distribution, *p* = .827 (Fig 3A). To estimate the reliability of congruency sequence effect, we performed a reliability analysis by calculating the split-half reliability coefficient for the size of congruency sequence effect. We firstly divided all trials into the odd and even trials, respectively. And then we calculated the size of congruency sequence effect in the odd and even trials, respectively. Lastly, the Guttman split-half coefficient was calculated. The results revealed that the split-half reliability coefficient was 0.68. To estimate the time course of congruency sequence effect, we conducted the RTs distribution analysis by using delta plots [45, 47, 48, 54, 63]. The results are showed in Fig 4A.

**Fig 5:**
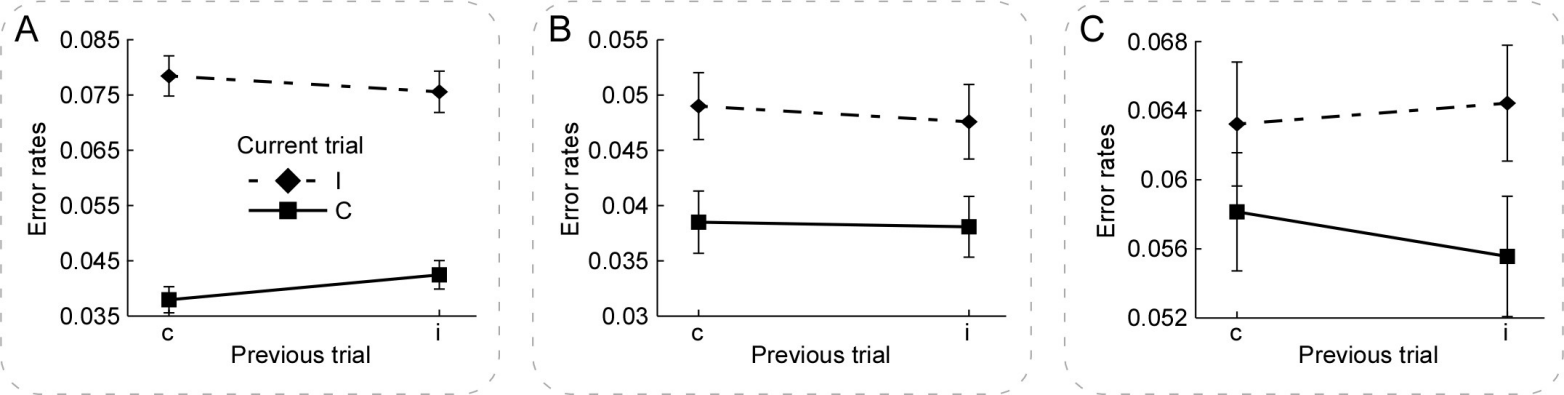
The error rates in Experiments 1, 2, & 3. Panels **A, B, & C** illustrate the error rates in the current trials, which were calculated according to the congruency (congruent, incongruent) in the previous and current trials in Experiments 1, 2, & 3, respectively. As shown in Panel **A**, the congruency effects in the current trials preceded by the congruent trials (cI–cC) were significantly larger than those preceded by the incongruent trials (iI–iC), which is accordant with the results of RTs. Panels **B & C** show comparable congruency effects in the current trials preceded by both the congruent and incongruent trials. For each condition, error bars represent ±SEM across the participants. NB. ‘c’ and ‘C’ are congruent condition and ‘i’ and ‘I’ are incongruent condition.

The generalized estimating equations were used to measure the error rates in the cC, cI, iC, and iI conditions with the following variables: the congruency (congruent, incongruent) in the previous trials, and the congruency (congruent, incongruent) in the current trials, of which age and gender were used as the covariates, which avoided the bias of spurious interaction in the estimation of the error rates [64]. The Bonferroni method was used when adjustment for multiple comparisons. The tests of model effects showed (1) significant main effect in the current trials, Wald Chi-Square = 205.30, *p* < .001; and (2) significant interaction effect between the congruency in the current trials and that in the previous trials, Wald Chi-Square = 4.43, *p* = .035. Pairwise comparison results showed significant lower error rates (1) in the cC relative to both cI and iI trials; and (2) in the iC relative to both cI and iI trials, each *p* < .001 (Bonferroni-corrected). The paired-samples T test showed that the congruency effects in the current trials preceded by the incongruent trials (iI–iC = 0.03±0.03) were significantly smaller than those preceded by the congruent trials (cI–cC = 0.04±0.04), *t*(121) *= -*2.10, *p* = .038, Cohen’s *d* = .244 [62]. Fig 5A illustrates the error rates in Experiment 1.

### Discussion

The present experiment found significant congruency sequence effect in the typical Stroop task. The results revealed typical pattern of congruency sequence effect in the current congruent condition, where the RTs were significantly shorter for the cC compared with iC condition in both the across-trial average level and single-trial level of each condition and participant (Tables 1 and 2). Importantly, the RTs_(iI–iC)_ were significantly smaller than the RTs_(cI–cC)_. These results replicated and extended several previous observations, i.e., congruency sequence effect was determined by the congruent condition in the current trials [21, 22, 65, 66]. Additionally, the differences of error rates in (iI–iC) were significantly smaller than those in (cI–cC), which are in accord with the results of RTs.

In the typical Stroop task, the target and distractor stimuli are processed in two separated pathway: the word reading and color naming pathways. The word reading is automatically processed while the color naming is controllably processed. The controlled process is related to task demand, and is affected by the automatic process. In this case, the word reading has a processing advantage over the color naming [55, 56] according to the parallel distributed processing model [57–59]. Hence, the smaller congruency effects preceded by the incongruent compared to congruent trials may be derived from that the processing advantage of word reading over the color naming is more enhanced in the cC relative to iC condition.

The distribution curve of congruency sequence effect did not significantly differ from a normal distribution (Fig 3A), which verified the inter-individual differences in congruency sequence effect. The Guttman split-half coefficient was 0.68, which suggested the moderate reliability of congruency sequence effect was [67]. Although other random factor may contribute to the inter-individual variability of congruency sequence effect, the Gaussian distribution of congruency sequence effect reflected some meaningful inter-individual variability. The congruency sequence effect increased over the slowest percentile bines in the delta plots (Fig 4A), suggesting the effective selective inhibition processes in these slowest trials and confirming the activation suppression hypothesis [48]. Thus, these results demonstrated the inter-trial and inter-individual variability of congruency sequence effect in the typical Stroop task.

Nevertheless, we also wonder if the pattern of variability of individual differences will be observed in other congruency tasks, where both the target and distractor are processed within the same pathway. Therefore, Experiment 2 adopted the word Flanker task [49, 50] to address the question and to expand the findings of Experiment 1. We used the experimental procedure and design that were same as those used in Experiment 1. In the word Flanker task, both target and flankers are processed in the same pathway and the target processing dimension always overlaps with the flanker processing dimension. The flanker processing dimension has no processing advantage over the target processing dimension according to the dimensional overlap theory [60, 61]. In this case, both the nature of task and processes of stimuli are different between the Stroop and word Flanker tasks. Thus, we anticipate the pattern of congruency sequence effect will differ from that in Experiment 1, and will be embodied in the current incongruent trials. However, we expect the other results will be consistent with those in Experiment 1 due to the same experimental design between two Experiments.

## Experiment 2

### Method

#### Participants

120 healthy right-handed volunteers (105 women), between 18 and 22 years old (19.82 ± 0.83, mean ± SD), took part in the experiment. All volunteers reported normal or corrected-to-normal vision and normal color perception. All volunteers provided written informed consent and were paid for their participation. The Zunyi Normal College Human Ethics Committee (Zunyi, China) approved the experimental procedure, which were in accordance with the standards of the Declaration of Helsinki. In addition, the volunteers were blind to the experimental design.

#### Apparatus, stimuli, and task

The apparatus were the same as those used in Experiment 1. The stimuli consisted of words (RED, GREEN, YELLOW, and BLUE; in Chinese with Song Ti font, size 20) that were printed in white font. In each trial, three horizontal-arranged words were presented in the center of the computer monitor with a central target word was flanked by a distractor word on each side. The distractor words were always identical to each other. The target word was either identical to the flanking words or different from them. For the congruent condition, the target and flankers were associated with the same response (e.g., “RED RED RED”); for the incongruent condition, the target and flankers were associated with the different responses (e.g., “GREEN RED GREEN”). Participants were instructed to response to the center word as quickly and accurately as possible, and to ignore the flanker words. They were instructed to press the “D” key using the left middle finger if the central word was “RED”, the “F” key using the left forefinger if the central word was “GREEN”, the “J” key using the right forefinger if the central word was “YELLOW”, and the “K” key using the right middle finger if the central word was “BLUE”. The rules of stimulus-response button mapping were counterbalanced across the participants.

#### Procedure and design

The experimental procedure (Fig 1B) and design were the same as those in Experiment 1.

### Results

The analyses of the RTs and error rates were the same as those in Experiment 1. For the analysis of the RTs, all incorrect trials (4.47% of all trials), post-error trials (4.02% of all trials), and RT outliers (±3 SDs, 1.24% of all trials) were excluded. Fig 2B illustrates the mean RTs in the current trials. Table 1 (the middle column) shows the two-way repeated-measures ANOVA results for the mean RTs. Table 2 (the middle column) shows the linear mixed model results for the RTs on the single-trial level of each condition of each participant, which are in accord with those of the mean RTs. The RTs_(iI–iC)_ (43.80±22.58) was significantly smaller than the RTs_(cI–cC)_ (50.61±22.64), *t*(119) = *-*2.88, *p* = .005, Cohen’s *d* = .302 [62].

We performed the normality test using the Shapiro-Wilk Test for RTs_[(cI–cC)–(iI–iC)]_. The results did not show significant difference from Gaussian distribution, *p* = .147 (Fig 3B). The Guttman split-half coefficient was calculated. The results revealed that the split-half reliability coefficient was 0.59. The time course of the congruency sequence effect was illustrated in Fig 4B.

The analyses of error rates were similar to those in Experiment 1. The tests of model effects only showed significant main effect in the current trials, Wald Chi-Square = 39.78, *p* < .001 (generalized estimating equations). The paired-samples T test showed that the congruency effects in the current trials preceded by the congruent trials (cI–cC = 0.01±0.02) was comparable with those preceded by the incongruent trials (iI–iC = 0.01±0.02), *t*(119) < 1, *p* = .717, Cohen’s *d* = .046 [62]. Fig 5B illustrates the error rates in Experiment 2.

### Discussion

In the present experiment, we expanded the findings of Experiment 1 in the word Flanker task. We found typical pattern of congruency sequence effect in the current incongruent condition, which is accord with the anticipation. That is, the RTs were significantly shorter for the iI compared to cI condition both in the across-trial average level and single-trial level of each condition and participant (Tables 1 and 2). More importantly, the RTs_(iI–iC)_ was significantly smaller than the RTs_(cI–cC)_. These results suggest that the RT patterns replicated and extended the previous observation, i.e., congruency sequence effect was determined by the incongruent condition in the current trials [14]. Altogether, these results provided empirical evidence verifying the conflict monitoring theory [5, 10] that congruency sequence effect stemmed from conflict-driven adjustments in cognitive control in the word Flanker task.

In addition, the present experiment found that the distribution curve of congruency sequence effect did not significantly differ from normal distribution (Fig 3B), which is consistent with the anticipation. The Guttman split-half coefficient was 0.59, which suggested the moderate reliability of congruency sequence effect [67]. Although other random factor may contribute to the inter-individual variability of congruency sequence effect, the Gaussian distribution of congruency sequence effect reflected some meaningful inter-individual variability. The congruency sequence effect increased over the slowest percentile bines in the delta plots (Fig 4B), suggesting the selective inhibition processes and confirming the activation suppression hypothesis [48]. These results are consistent with the findings of Experiment 1. Taken together, the findings of Experiments 1 & 2 demonstrate the inter-trial and inter-individual variability of congruency sequence effect across the Stroop and word Flanker tasks.

It is notable that both the target and flankers are processed in the word reading pathway in the word Flanker task, and the flanker processing dimension has no processing advantage over the target processing dimension [60, 61]. In this case, the nature of task is different from that of Stroop task. As the processing of semantics is implicated in some complicated cognitive processes, the processes of stimuli in the word Flanker task are also different from those in the letter Flanker task. Thus, although the nature of word Flanker task is same as that of letter Flanker task, the levels of processes of stimuli are different between tasks. However, it is unclear whether the present findings can be expanded to the letter Flanker task, in which the target processing dimension always overlaps with that of the flankers, and both the target and flankers are processed in the letter level.

Hence, in Experiment 3, we used the letter Flanker task to investigate the inter-trial and inter-individual variability of congruency sequence effect. As the processes of stimuli are different from those in the Stroop and word Flanker tasks, we anticipate the pattern of congruency sequence effect, the characteristics of Gaussian distribution, and the time course of congruency sequence effect will differ from those in Experiments 1 & 2.

## Experiment 3

### Method

#### Participants

124 healthy right-handed volunteers (109 women), between 18 and 24 years old (20.23 ± 1.09, mean ± SD), took part in the experiment. All volunteers reported normal or corrected-to-normal vision and normal color perception. All volunteers provided written informed consent and were paid for their participation. The Zunyi Normal College Human Ethics Committee (Zunyi, China) approved the experimental procedure, which were in accordance with the standards of the Declaration of Helsinki. In addition, the volunteers were blind to the experimental design.

#### Apparatus, stimuli, and task

The apparatus were the same as those used in Experiments 1 & 2. The stimuli consisted of capital letters (K, N, F, and W; in Times New Roman font, size 20) that were printed in white font. In each trial, horizontal-arranged capital letters were presented in the center of the computer monitor with a central target letter was flanked by two distractor letters on each side. The distractor letters were always identical to each other. The target letter was either identical to the flanking letters or different from them. For the congruent condition, the target and flankers were associated with the same response (e.g., “KKKKK”); for the incongruent condition, the target and flankers were associated with the different responses (e.g., “NNFNN”).

Participants were instructed to response to the center word as quickly and accurately as possible, and to ignore the flanker words. They were instructed to press the “1” key using the left middle finger if the central letter was “K”, the “2” key using the left forefinger if the central word was “N”, the “9” key using the right forefinger if the central word was “F”, and the “0” key using the right middle finger if the central word was “W”. The rules of stimulus-response button mapping were counterbalanced across the participants.

#### Procedure and design

The experimental procedure (Fig 1C) and design were the same as those in Experiment 1.

### Results

The analyses of the RTs and error rates were the same as those in Experiments 1 & 2. For the analysis of the RTs, all incorrect trials (6.15% of all trials), post-error trials (5.54% of all trials), and RT outliers (±3 SDs, 1.12% of all trials) were excluded. Fig 2C illustrates the mean RTs in the current trials. Table 1 (the right column) shows the two-way repeated-measures ANOVA results for the mean RTs. Table 2 (the right column) shows the linear mixed model results for the RTs on the single-trial level of each condition of each participant, which are in accord with those of the mean RTs. In addition, the RTs_(iI–iC)_ (28.11±22.77) was not significantly different from the RTs_(cI–cC)_ (28.24±26.98), *t*(123) < 1, *p* = .960, Cohen’s *d* = .057 [62].

We performed the normality test using the Shapiro-Wilk Test for RTs_[(cI–cC)–(iI–iC)]_. The results did not show significant difference from Gaussian distribution, *p* = .269 (Fig 3C). The Guttman split-half coefficient was calculated. The results revealed that the split-half reliability coefficient was 0.19. The time course of the congruency sequence effect was illustrated in Fig 4C.

The analyses of error rates were similar to those in Experiments 1 and 2. The tests of model effects only showed significant main effect in the current trials, Wald Chi-Square = 12.37, *p* < .001 (generalized estimating equations). The paired-samples T test showed that the congruency effects in the current trials preceded by the congruent trials (cI–cC = 0.01±0.03) was comparable with those preceded by the incongruent trials (iI–iC = 0.01±0.03), *t*(123) *=* 1.16, *p* = .249, Cohen’s *d* = .126 [62]. Fig 5C illustrates the error rates in Experiment 3.

### Discussion

In the present experiment, we investigated the inter-trial and inter-individual variability of congruency sequence effect in the letter Flanker task. The results showed non-significant interactions between the congruency in the previous and that in the current trials for both the RT (Tables 1 and 2) and error rate data. Additionally, the congruency effects in the current trials preceded by the congruent trials were comparable with that preceded by the incongruent trials for both the mean RT and error rate data. Therefore, the results of Experiment 3 indicate a non-significant congruency sequence effect in the letter Flanker task. Although the distribution curve of congruency sequence effect did not significantly differ from normal distribution (Fig 3C), the Guttman split-half coefficient was 0.09, which suggested the poor reliability of congruency sequence effect [67] and the Gaussian distribution of congruency sequence effect reflect some measurement or random noise. Maybe other factors also influence the reliability of the Gaussian distribution, which should be discussed in the future. The congruency sequence effect decreased over the slowest percentile bines in the delta plots (Fig 4C), which was not in accord with the results of Experiments 1 & 2. Maybe both the nature and difficult of task explained these findings. Altogether, these results showed that congruency sequence effect was very weak and the inter-trial and inter-individual variability of congruency sequence effect might reflect some unmeaning random noise in the letter Flanker task.

Combined with the findings of Experiments 1 & 2, these findings supported for our speculation that the pattern of congruency sequence effect, the characteristics of Gaussian distribution, and the time course of congruency sequence effect in Experiment 3 differed from those in Experiments 1 & 2. It is notable that the experimental design was the same across all three Experiments. The only difference was the experimental task. Thus, the findings of Experiments 1, 2, & 3 demonstrate that the inter-trial and inter-individual variability of congruency sequence effect depends on the nature of tasks.

## General discussion

The present study investigated the variability of individual differences in congruency sequence effect during performances of the Stroop, word Flanker, and letter Flanker tasks in three experiments, respectively. To get the pure measure of congruency sequence effect, the confounding of both the bottom-up repetition effects [51, 52] and negative priming effects [53] was excluded across the three experiments. It is notable that the proportion of the congruent and incongruent trials was equal, and the experimental design was the same in all three Experiments. We obtain distinct patterns of congruency sequence effect in all three Experiments. The congruency sequence effect was embodied in the congruent and incongruent trials in the Stroop and word Flanker tasks, respectively, while it was absent in the letter Flanker task (Tables 1 and 2, Fig 2). The linear mixed model results of the single-trial RTs are in accord with the repeated-measures ANOVA results of the mean RTs. Additionally, the results consistently show that the distributional properties of congruency sequence effect were not significant different from the Gaussian distribution in the Stroop and word Flanker tasks, but not in the letter Flanker task (Fig 3). The delta plots (Fig 4) reveal that congruency sequence effect increased over the slowest quantile bines in the Stroop and word Flanker tasks, which verifies the activation suppression hypothesis [48]; however, it decreased with the increasing mean RTs in the letter Flanker task, suggesting the poor reliability of congruency sequence effect. These combined effects are likely explained by the differences in both the nature of task and the levels of processes of stimuli. Therefore, the present study provides appealing evidence verifying a task-dependent characteristic of congruency sequence effect, and revealing the inter-trial and inter-individual variability of cognitive control. Taken together, these findings are consistent with our assumption, which the variability of individual differences in congruency sequence effect are task-dependent, and support for the conflict monitoring theory [5, 10].

The conflict-monitoring model proposes that congruency sequence effect stems from conflict-driven adjustments in cognitive control [10]. Accordingly, the occurrence of conflict activates the cognitive control system, and the information of conflict is detected by the ACC, which alerts the DLPFC to implement an up-regulation in cognitive control to resolute the conflict. Therefore, the level of cognitive control is high during trials following an incongruent trial, e.g., iI and iC trials. In contrast, if there are no the occurrence of conflict, the up-regulation of cognitive control will be absent. Thus, the level of control is low during trials following a congruent trial, e.g., cI and cC trials. The increased cognitive control results in a stronger attentional biasing of information processing in line with the task demands in the current trial. In this case, participants may adopt a highly attentional focused manner to process the stimulus, which resulting in that the processing of target is enhanced [13], and the effect of the distractor on performance is reduced. These combined effects result in the decreased RTs in the iI compared to cI trial. In addition, the absence of conflict in the previous trial will result in a decrease in cognitive control. In this case, the processing of distractor is enhanced. In the cC trial, the enhancement in the processing of distractor effectively facilitates the processing of congruent stimulus. Therefore, the RTs are reduced in the cC compared with iC trial.

In Experiment 1, we found that (1) the RTs were significantly slower for the iC relative to cC condition, and (2) the RTs_(iI–iC)_ were significantly smaller than the RTs_(cI–cC)_ in the Stroop task (Fig 2A). In Experiment 2, we found that (1) the RTs were significantly shorter for the iI relative to cI condition, and (2) the RTs_(iI–iC)_ were significantly smaller than the RTs_(cI–cC)_ in the word Flanker task (Fig 2B). These results show that congruency sequence effect was determined by the congruent and incongruent conditions in the Stroop and word Flanker tasks, respectively (Fig 2A & 2B), which expanded the previous studies demonstrating that congruency sequence effect was determined by either the congruent condition [11, 21, 22, 65, 66, 68] or the incongruent condition [14, 69]. According to the conflict-monitoring theory [5, 10], in the Stroop task, congruency sequence effect may be derived from an enhanced facilitation of the processing of the cC trials or a reduced facilitation of the processing of the iC trials; in the word Flanker task, congruency sequence effect may be derived from a reduced interference of the processing of the iI trials or an increased interference of the processing of the cI trials.

In Experiment 3, the congruency sequence effect was absent (Fig 2C). In the Stroop task, the target dimension of the stimulus is the printed color, the distractor dimension of the stimulus is the word semantics. The two dimensions are separately processed in color naming and word reading pathways, and the word reading has a processing advantage over the color naming [55, 56] according to the parallel distributed processing model [57–59]. In the word Flanker and letter Flanker tasks, both the target and distractor dimensions of the stimulus are the words (for the word Flanker task) or letters (for the letter Flanker task). The target and distractor are processed in the same pathway, and the distractor dimensions of the stimulus has no processing advantage over the target dimension of the stimulus according to the dimensional overlap theory [60, 61]. However, the levels of processes of stimuli are distinct in the word Flanker and letter Flanker tasks, resulting in the differences in difficulty of tasks. In this case, both the nature and difficulty of tasks are different in all three tasks. As proposed by Botvinick et al. [8], conflict may serve as an index of the demand for mental effort [70, 71]. Accordingly, the absent of congruency sequence effect in the letter Flanker task may indicate that participants engaged in less effortful cognitive control processing due to that the difficulty of task was not enough [72]. As shown in Fig 2, the congruency effects were indeed smaller in the letter Flanker relative to both Stroop and word Flanker task and, therefore, the cognitive control processing was less effortful and the trial-by-trial adaptation was smaller as well and less statistically reliable [72]. Maybe this conclusion needs to be confirmed in future researches. Taken together, we consider that the difficulty of task is not enough in the letter Flanker task, resulting in the absence of congruency sequence effect. Thus, our results suggest a task-dependent characteristic of congruency sequence effect in cognitive control.

It is notable that (1) the distributional properties of congruency sequence effect were not significantly different from the Gaussian distribution and (2) the reliability coefficient of congruency sequence effect were moderate in both the Stroop and word Flanker tasks (Fig 3), suggesting some reliable and meaningful inter-individual variability of congruency sequence effect in the Stroop and word Flanker task. Additionally, although the distributional properties of congruency sequence effect were not significantly different from the Gaussian distribution in the letter Flanker task, the reliability coefficient of congruency sequence effect was poor, which suggest the reliability of the Gaussian distribution was influenced by random or measuring noise in the letter Flanker task. Considering the Gaussian distribution model may demonstrate typical inter-individual differences, the converging results reveal the inter-individual variability of congruency sequence effect depends on the nature of tasks. Therefore, the present study deepens our understanding of the distributional characteristics of congruency sequence effect in the Stroop, word Flanker, and letter Flanker tasks.

Additionally, as shown in Fig 4, the time course of congruency sequence effect (as expressed in RTs_[(cI–cC)–(iI–iC)]_), RTs_[(cI–cC)]_, and RTs_[(iI–iC)]_ pronouncedly differed in the Stroop, word Flanker, and letter Flanker tasks. In the Stroop task, congruency sequence effect, RTs_[(cI–cC)]_, and RTs_[(iI–iC)]_ increased across the entire RTs distribution. Specifically, congruency sequence effect showed a sharp increase in the last three quantiles. As congruency sequence effect was determined by the congruent condition, the increased congruency sequence effect and RTs_[(cI–cC)]_ over the slowest bins verify the activation suppression hypothesis [48], which proposes that inhibition takes time to accumulate and, therefore, more effective in trials with slower relative to faster RTs. In the word Flanker task, congruency sequence effect increased over the last quantile bines, which is accord with the findings in Experiment 1. However, RTs_[(iI–iC)]_ decreased over the slowest quantile bines. As congruency sequence effect was determined by the incongruent condition, the increased congruency sequence effect and decreased RTs_[(iI–iC)]_ over the slowest bins may suggest an effective selective inhibition process with the lengthened mean RTs [48]. In the letter Flanker task, congruency sequence effect decreased over the five bines, especially in the last two quantile bines, which may be accounted for the poor and unreliable congruency sequence effect. Thus, these results reveal the inter-trial and inter-individual variability of congruency sequence effect, and verify the task-dependent characteristic of congruency sequence effect in cognitive control.

## Conclusion

In three experiments, the present study finds some interesting results and extends previous work regarding the inter-trial and inter-individual variability of congruency sequence effect in the Stroop, word Flanker, and letter Flanker tasks. The results consistently manifest (1) the task-dependent distributional characteristic of congruency sequence effect and (2) the inter-trial and inter-individual variability of congruency sequence effect in the Stroop, word Flanker, and letter Flanker task. As such, the present work provides an important step toward understanding the essence of congruency sequence effect in the field of cognitive control.
